# Protocol for Efficient CRISPR/Cas9/AAV-Mediated Homologous Recombination in Mouse Hematopoietic Stem and Progenitor Cells

**DOI:** 10.1016/j.xpro.2020.100028

**Published:** 2020-06-03

**Authors:** Ngoc Tung Tran, Janine Trombke, Klaus Rajewsky, Van Trung Chu

**Affiliations:** 1 Immune Regulation and Cancer, Max-Delbrück-Center for Molecular Medicine in the Helmholtz Association, Berlin 13125, Germany

## Abstract

Mutations that accumulate in self-renewing hematopoietic stem and progenitor cells (HSPCs) can cause severe blood disorders. To model such disorders in mice, we developed a CRISPR/Cas9/adeno-associated virus (AAV)-based system to knock in and repair genes by homologous recombination in mouse HSPCs. Here, we provide a step-by-step protocol to achieve high efficiency of gene knockin in mouse HSPCs, while maintaining engraftment capacity. This approach enables the functional study of hematopoietic disease mutations *in vivo*, without requiring germline mutagenesis.

For complete details on the use and execution of this protocol, please refer to Tran et al. (2019).

## Highlights

• AAV particle production for efficient homologous recombination
• Isolation and *in vitro* culture of mouse HSPCs for gene knockin
• CRISPR/Cas9/AAV-mediated gene knockin in mouse HSPCs

## Graphical Abstract

**Figure:**
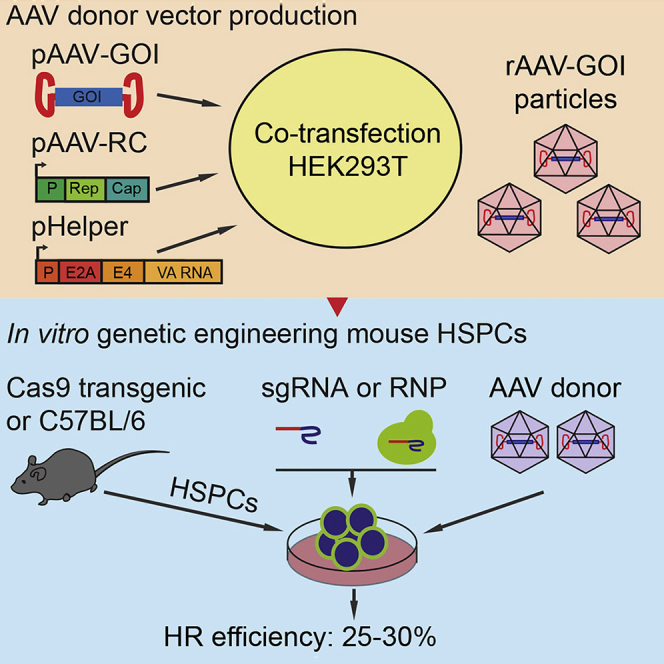

## BEFORE YOU BEGIN

### Experimental Design Considerations

**Timing: 1–2 weeks**

1. To achieve efficient homologous recombination (HR) events in mouse HSPCs, a specific single-guide RNA (sgRNA) with high editing efficiency is required. We use the CrispRGold program (https://crisprgold.mdc-berlin.de) to design specific sgRNAs and to predict potential off-targets (Chu et al., 2016a). Several specific sgRNAs should be designed per targeted sequence. Editing efficiencies of all sgRNAs must be validated by measuring mismatched DNA heteroduplexes using the T7 endonuclease I assay (Guschin et al., 2010) and Sanger sequencing of PCR products for at least 2 primary blood cell types, such as B and T cells. sgRNAs can be ordered as chemically modified or unmodified forms from IDT, Synthego or alternative suppliers.
2. The optimal design of the donor template is essential for efficient HR in mouse HSPCs. The donor template includes 5’, 3’ homology arms and desired modified gene sequences. The length of the homology arms is dependent on the specificity of the targeted sequences and consists of 600 to 2000 bp, each. The packaging capacity of the AAV genome is a limitation for designing the donor template as the maximal length of AAV-based donor templates should not exceed 4.5kb. In case no reporter gene is used, a restriction enzyme recognition site should be added to the modified gene sequences through introducing silent mutations that can be used to quantify HR efficiency.
3. In order to quantify HR and non-homologous end joining (NHEJ) events in the targeted loci by PCR amplification and sequencing, forward or reverse primers, or both must be designed outside of the 5’ or 3’ homology arms. Primer sets outside of the homology arms should exclude the possibility of donor template amplification. For amplifying large PCR fragments (>2.0 kb), we use LongAmp Polymerase.
4. Mouse strains: We have successfully performed reporter knockins in HSPCs isolated either from C57BL/6 or Cas9 transgenic mice as reported previously (Chu et al., 2016b). The targeted HSPCs can be transplanted into sublethally irradiated Rag2^−/−^cγ^−/−^ (Tran et al., 2019) or lethally irradiated immunocompetent mice (Duran-Struuck et al., 2008) to assess engraftment efficiency of the targeted HSPCs.
5. Healthy and low passage HEK293T cells are critical for a high yield of AAV. The HEK293T cells used for AAV production are maintained below 80% confluence and passaged less than 15 times. After thawing, HEK293T cells should be kept in culture for at least one passage before seeding for AAV production.

## KEY RESOURCES TABLE

**Table:** 

REAGENT or RESOURCE	SOURCE	IDENTIFIER
**Antibodies**		

TrueStain FcΧ™ (anti-mouse CD16/32, clone 93)	BioLegend	Cat#101320 RRID:AB_1574973
BV785 anti-mouse Ly6A/E (Sca1) (clone D7)	BioLegend	Cat#108139 RRID:AB_2565957
APC anti-mouse CD117 (c-Kit) (clone 2B8)	BioLegend	Cat#105812 RRID:AB_313221
APC/Cy7 anti-mouse CD48 (clone HM48-1)	BioLegend	Cat#103431 RRID:AB_2561462
BV605 anti-mouse CD150 (clone TC15-12F12.2)	BioLegend	Cat#115927 RRID:AB_11204248
PE/Cy7 anti-mouse B220 (clone RA3-6B2)	BioLegend	Cat#103221 RRID:AB_313004
PE/Cy7 anti-mouse CD3e (clone 145-2C11)	BioLegend	Cat#100319 RRID:AB_312684
PE/Cy7 anti-mouse Gr-1 (clone RB6-8C5)	BioLegend	Cat#108415 RRID:AB_313380
PE/Cy7 anti-mouse Ter-119 (clone TER-119)	BioLegend	Cat#116221 RRID:AB_2137789
PE/Cy7 anti-mouse CD11b (clone M1/70)	BioLegend	Cat#101215 RRID:AB_312798
PE/Cy7 anti-mouse CD11c (clone N418)	BioLegend	Cat# 117317 RRID:AB_493569

**Bacterial and Virus Strains**		

TOP10	Invitrogen	Cat#C404003
AAV-DJ	Cell Biolabs	Cat#VPK-400-DJ

**Biological Samples**		

Mouse primary cells	MDC Berlin, Germany	N/A

**Chemicals, Peptides, and Recombinant Proteins**		

DAPI	Sigma Aldrich	Cat#D9542
Sodium azide (NaN_3_)	Sigma Aldrich	Cat#S8032-25G
Sodium chloride (NaCl)	Carl Roth	Cat#3957.2
Potassium bicarbonate (KHCO_3_)	Sigma Aldrich	Cat# 237205
Potassium chloride (KCl)	Sigma Aldrich	Cat#60128-1Kg-F
DOC (sodium deoxycholate)	Sigma Aldrich	Cat#D6/50-25G
Magnesium chloride (MgCl_2_)	ROTH	Cat#KK363
Sodium succinate( C_4_H_4_Na_2_O_4_)	SERVA (Germany)	Cat#14972
EDTA	ROTH	Cat#8040.2
Sodium phosphate monobasic (NaH_2_PO_4_)	Sigma Aldrich	Cat# S8282
Sodium phosphate dibasic (Na_2_HPO_4_)	Sigma Aldrich	Cat# S7907
Tris-HCl	ROTH	Cat#9090.3
Agarose	Biozym	Cat#840004
Ethidium bromide solution	Invitrogen	Cat#15585-011
Benzonase Nuclease 250 U/μL	Millipore	Cat#:71205-3
RNase-free DNase I	Qiagen	Cat#:79254
SpCas9 protein	MDC Berlin, Germany	N/A
SpCas9 protein	IDT	Cat#1081059
99% Ethanol	MDC Berlin, Germany	N/A
StemSpan™ SFEM II	Stem Cell Technologies	Cat#09655
DMEM^+/+^	Gibco	Cat#41966052
Opti-MEM	Gibco	Cat# 31985047
1 M HEPES	Gibco	Cat#15630056
PBS pH 7.2	Gibco	Cat#20012019
BSA, Albumin Fraction V	Carl Roth	Cat#8076.3
FBS	Sigma Aldrich	Cat#F7524-500mL
Gentamycin	Lonza	Cat#17-519L
OptiPrep™ Density Gradient Medium	Sigma Aldrich	Cat#D1556-250mL
Mouse recombinant TPO	Peprotech	Cat#315-14
Mouse recombinant SCF	Peprotech	Cat#250-03
Mouse recombinant Flt3-ligand	Peprotech	Cat#250-31L
Human recombinant IL-11	Peprotech	Cat#200-11
Quick Extract™ DNA Extraction Solution 1.0	Lucigen	Cat#QE09050
Phenol Red solution	Sigma Aldrich	Cat#P0290
10% Pluronic® F-68	Gibco	Cat#24040-032
Polyethylenimine (PEI)	Polysciences	Cat#23966-1
Trysin-EDTA	Sigma	Cat#T3924-100mL

**Critical Commercial Assays**		

Anti-mouse Sca1 MicroBead Kit (VioBright™ FITC)	Miltenyi Biotec	Cat#130-123-124
LongAmp PCR Master Mix	New England Biolabs	Cat#M0533L
KOD Hot Start DNA Polymerase	Millipore	Cat#71086-3
p.JET TOPO cloning Kit	Thermo Fischer	Cat#K1231
T7 endonuclease I	NEB	Cat#M0302S
CellTrace™ Violet	Invitrogen	Cat#C34557
TaqMan Universal PCR MasterMix	Applied Biosystems	Cat#4324018

**Experimental Models: Cell Lines**		

HEK293T	ATCC	ATCC®CRL-3216

**Experimental Models: Organisms/Strains**		

C57BL/6	Taconic	C57BL/6NTac
R26-Cas9iGFP	Chu et al., 2016b	N/A
Rag2^−/−^cγ^−/−^	Taconic	N/A

**Oligonucleotides**		

Chemically modified and unmodified sgRNAs	Synthego or IDT	N/A
AAV ITR primers FOR 5′-CGGCCTCAGTGAGCGA-3′ REV 5′-GGAACCCCTAGTGATGGAGTT-3′	Eurofins	N/A
AAV ITR probe 5′-CACTCCCTCTCTGCGCGCTCG-3′	Eurofins	N/A

**Recombinant DNA**		

AAV-DJ Rep/Cap plasmid	Cell Biolabs	Cat#VPK-400-DJ
AAV-helper plasmid	Cell Biolabs	Cat#VPK-400-DJ
pAAV-DJ plasmid	Cell Biolabs	Cat#VPK-400-DJ
pAAV-mCherry genome plasmid	Tran et al., 2019	N/A

**Software and Algorithms**		

FlowJo	LLC	https://www.flowjo.com/
CrispRGold	Chu et al., 2016a	https://crisprgold.mdc-berlin.de
ImageJ	NIH imageJ	https://imagej.nih.gov/ij

**Other**		

Dry ice	MDC Berlin, Germany	N/A
PCR Stripe Tubes	Axygen	Cat#PCR-0208-CPC
MicroAmp Fast 96-well reaction plate (0.1 mL)	Applied Biosystems	Cat#4346907
MicroAmp Clear Adhesive Film	Applied Biosystems	Cat#4306311
Beckmann Optima L-80 XP Ultracentrifuge	Beckmann Coulter	N/A
StepOnePlus Real-time PCR system	Applied Biosystems	N/A
Mastercycler® nexus X2	Eppendorf	N/A
Amaxa 4D Nucleofector	Lonza	N/A
16-well Nucleocuvette™ Strips	Lonza	Cat#V4XP-3032
BD Filcon, Sterile, Cup-Type	BD Biosciences	Cat#340629
Type 70 Ti Fixed-Angle Titanium Rotor	Beckmann Coulter	Cat#337922
OptiSeal tube adapters	Beckmann Coulter	Cat#361669
150 mm petri dishes	Sarstedt	Cat#82.1473
50 mL Polystyrene Conical Tube	Corning	Cat#352070
15 mL Polystyrene Conical Tube	Corning	Cat#352096
Optiseal Polypropylene Centrifugation Tubes	Beckmann Coulter	Cat#361625
Amicon® Ultra -15 Centrifugal Units-100K	Merck Millipore	Cat#UFC910008
Slide-A-Lyzer® Dialysis Cassette 10.000 MWCO	Thermo Scientific	Cat#66811
0.45 μm PES filter	Jet Biofill	Cat#FCA-406-030
0.22 μm PES filter small	Santa Cruz Biotechnology	Cat#Sc-516079
0.22 μm PES filter	Whatman Life Sciences	Cat#10462200
50 μm Filter mesh	BD	Cat#340632
2 10 × 80 mm 14G × 3 1/8 inch needle	Braun	Cat#4665473
1.2 38 mm 18Gx 1 ½ inch needle	Terumo®	Cat#AN∗1838S1
0.9 × 40 mm 20G 1 ½ inch needle	Braun	Cat#4657519
0.5 × 16 mm 25G 5/8 inch needle	Henry Schein®	Cat#9003629
0.4 × 19 mm 27G 1 ½ inch needle	BD	Cat#302200
1 mL syringe	Braun	Cat#9161406V
5 mL syringe	Braun	Cat#4606051V
10 mL syringe	Braun	Cat#4606108V
20 mL syringe	Braun	Cat#4606205V
Kimwipe Tissue	Kimwipe	Cat#7552
Cell Scraper 245 mm length	Carl Roth	Cat#EKX9.1
LS Columns	Miltenyi Biotec	Cat#130-042-401
MS Columns	Miltenyi Biotec	Cat#130-042-201

## MATERIALS AND EQUIPMENT

**Table:** Red Blood Cell Lysis Buffer

Reagent	Final Concentration (mM)	Volume
NHCl_4_ (powder)	155	8.3 g
KHCO3 (powder)	10	1 g
EDTA (powder)	0.1	0.037 g
ddH_2_O	n/a	1 L
**Total**	**n/a**	**1 L**

Mix to dissolve and adjust pH to 7.5 with HCl. Autoclave and store this solution at 4°C for 6 months.

**Table:** FACS Buffer

Reagent	Final Concentration (%)	Volume
BSA (powder)	1	5 g
NaN_3_ (10%)	0.09	4.5 mL
PBS (pH 7.2)	n/a	500 mL
**Total**	**n/a**	**500 mL**

Mix well and sterile filter. Store this solution at 4°C for 3 months.

**Table:** Amaxa Electroporation Buffer

Reagent	Final Concentration (mM)	Volume (mL)
NaH_2_PO_4_ (1 M)	25.2	0.252
Na_2_HPO_4_ (1 M)	64.8	0.648
KCl (1 M)	5	0.05
MgCl_2_ (1 M)	10	0.1
HEPES (200 mM)	20	1
C_4_H_4_Na_2_O_4_ (240 mM)	24	1
ddH_2_O	n/a	6.96
**Total**	**n/a**	**10**

Mix well in the hood and sterile filter. Store this solution at 4°C for 3 months.

**Table:** Polyethyleneimine (PEI) Solution

Reagent	Final Concentration (mg/mL)	Volume
PEI (powder)	1	500 mg
ddH_2_O	n/a	500 mL
**Total**	**n/a**	**500 mL**

Mix to dissolve at 75°C in a water bath, cool down to 25°C and adjust pH 7.0 with HCl. Aliquot and store this solution at -80°C.

**Table:** Dialysis/Concentration Buffer

Reagent	Final Concentration	Volume (mL)
NaCl (5 M)	50 mM	5
10% Pluronic F68	0.0001%	0.05
PBS (pH 7.2)	n/a	500
**Total**	**n/a**	**500**

Mix well in the hood and sterile filter. Store this solution at 22°C–24°C for 6 months.

**Table:** Balancing Buffer

Reagent	Final Concentration (M)	Volume (mL)
Tris-HCl pH 8.5 (1 M)	0.01	5
NaCl (5 M)	1	100
PBS (pH 7.2)	n/a	500
**Total**	**n/a**	**500**

Mix well in the hood and sterile filter. Store this solution at 22°C–24°C for 6 months.

**Table:** PBS-MgK Buffer

Reagent	Final Concentration (mM)	Volume (mL)
MgCl_2_ (1 M)	1	0.5
KCl (1 M)	2.5	1.25
PBS (pH 7.2)	n/a	500
**Total**	**n/a**	**500**

Mix well in the hood and sterile filter. Store this solution at 22°C–24°C for 6 months.

**Table:** PBS-MgKNa Buffer

Reagent	Final Concentration (M)	Volume
NaCl (powder)	1	29.22 g
PBS-MgK buffer	n/a	500 mL
**Total**	**n/a**	**500 mL**

Mix well in the hood and sterile filter. Store this solution at 22°C–24°C for 6 months.

**Table:** 15% Iodixanol

Reagent	Final Concentration (%)	Volume (mL)
OptiPrep	15	7.5
PBS-MgKNa buffer	n/a	22.5
**Total**	**n/a**	**30**

**Table:** 25% Iodixanol

Reagent	Final Concentration (%)	Volume (mL)
OptiPrep	25	12.5
PBS-MgK buffer	n/a	17.5
Phenol Red	0.25	0.075
**Total**	**n/a**	**30**

**Table:** 40% Iodixanol

Reagent	Final Concentration (%)	Volume (mL)
OptiPrep	40	14.7
PBS-MgKNa buffer	n/a	7.3
**Total**	**n/a**	**22**

**Table:** 60% Iodixanol

Reagent	Final Concentration (%)	Volume (mL)
OptiPrep	60	22
Phenol Red	0.45	0.1
**Total**	**n/a**	**22**

**CRITICAL:** Sodium Azide (NaN_3_) is toxic to aquatic life with long lasting effects. Carefully handle this chemical reagent following safety instruction of the manufacturer.

## STEP-BY-STEP METHOD DETAILS

### AAV Production

**Timing: 5 days**

Transfect, harvest and lyse transfected HEK293T cells for AAV production.

1. Maintain HEK293T cells in a 150-mm cell culture dish containing DMEM^+/+^ (DMEM with high glucose and sodium pyruvate) supplied with 10% FBS (HEK293T medium) below 80% confluence and harvest at around 80% confluence.
2. Day 0: Wash cells with PBS and trypsinize with Trypsin-EDTA for 5 min at 37°C. Collect cells by adding 10 mL of fresh HEK293T medium and pipetting up and down 10 times. Centrifuge at 290 × g for 5 min at 4°C and discard the supernatant. Resuspend the cell pellet in 20 mL HEK293T medium and count cell number using a hemocytometer. Plate 1 × 10^7^ cells in a 150-mm dish in 20 mL HEK293T medium. For each AAV donor vector, prepare eight 150-mm dishes.
3. Day 1: 2 h before transfection, aspirate the old medium and add 15 mL of fresh HEK293T medium containing 25 mM HEPES (add 12.5 mL of 1 M HEPES per 500 mL HEK293T medium) to each 150-mm dish containing the HEK293T cells.
a) Per 150-mm dish, prepare a transfection mixture as follows: 2 mL 22°C–24°C Opti-MEM + 10 μg pAAV genome + 10 μg pCap/Rep-DJ + 20 μg pAAV-helper plasmids. Gently mix by vortexing and slowly add 120 μL PEI (1 mg/mL) to the transfection mixture at a 3:1 ratio of PEI (μg): DNA (μg). Vortex briefly and incubate at 22°C–24°C for 10 min.
b) Add the transfection mixture dropwise to each 150-mm dish. Mix gently and transfer the dishes to a cell incubator. Culture the transfected cells at 37°C, 5% CO_2_.
4. Day 2: Aspirate the old medium and add 25 mL of fresh HEK293T medium to each transfected 150-mm dish. Culture the transfected cells until day 4 for AAV collection.
5. Day 4: Collect the transfected cells using a cell scraper. Transfer medium and cells from two of the transfected 150-mm dishes to a 50 mL collection tube using a serological 25 mL pipette. Centrifuge at 2900 × g for 5 min at 4°C and discard the supernatant. Resuspend the cell pellet in 3 mL PBS and transfer to a 15 mL collection tube.

**CRITICAL:** The transfection efficiency of three plasmids should be regularly assessed with a pAAV-CMV-GFP-reporter genome plasmid as a standard.

***Note:*** To avoid bacterial contamination after transfection, we recommend adding Gentamicin to a final concentration of 10 μg/mL in HEK293T medium used on day 2.

**Pause Point:** The cell suspension can be frozen and stored at -80°C for later AAV purification.

6. Prepare a dry ice/ethanol mixture in a foam box and heat up the water bath to 37°C.
7. Use three freeze-thaw cycles (10 min in the dry ice/ethanol mixture, then move to 37°C water bath for 10 min) to lyse the cells and release the AAV particles. Vortex the cell suspension thoroughly after each freeze-thaw cycle.
8. After the third freeze-thaw cycle, add Benzonase enzyme to a final concentration of 100 U/mL and incubate for 1 h at 37°C to degrade residual DNA plasmids in the cell lysate. Mix by inverting occasionally.
9. Add sodium deoxycholate (10% stock solution) to a final concentration of 0.5%. Add 5 M NaCl to a final concentration of 1 M. Incubate for 30 min at 37°C.
10. Centrifuge at 2900 × g for 30 min at 4°C. Transfer the viral supernatant to a 15 mL collection tube.
11. Filter the AAV supernatant through a 0.45 μm PES syringe filter.
12. Finally, pool all the filtered AAV supernatants into a 50 mL collection tube (~15 mL in total).

**CRITICAL:** Ensure that the cell suspension is completely frozen and thawed after each step.

**Pause Point:** The AAV supernatant can be stored at 4°C for 15–18 h and the ultra-centrifugation performed the next day.

### AAV Purification

**Timing: 4 h**

Prepare iodixanol-containing gradient, ultra-centrifuge and extract AAV particles.

**Figure 1:**
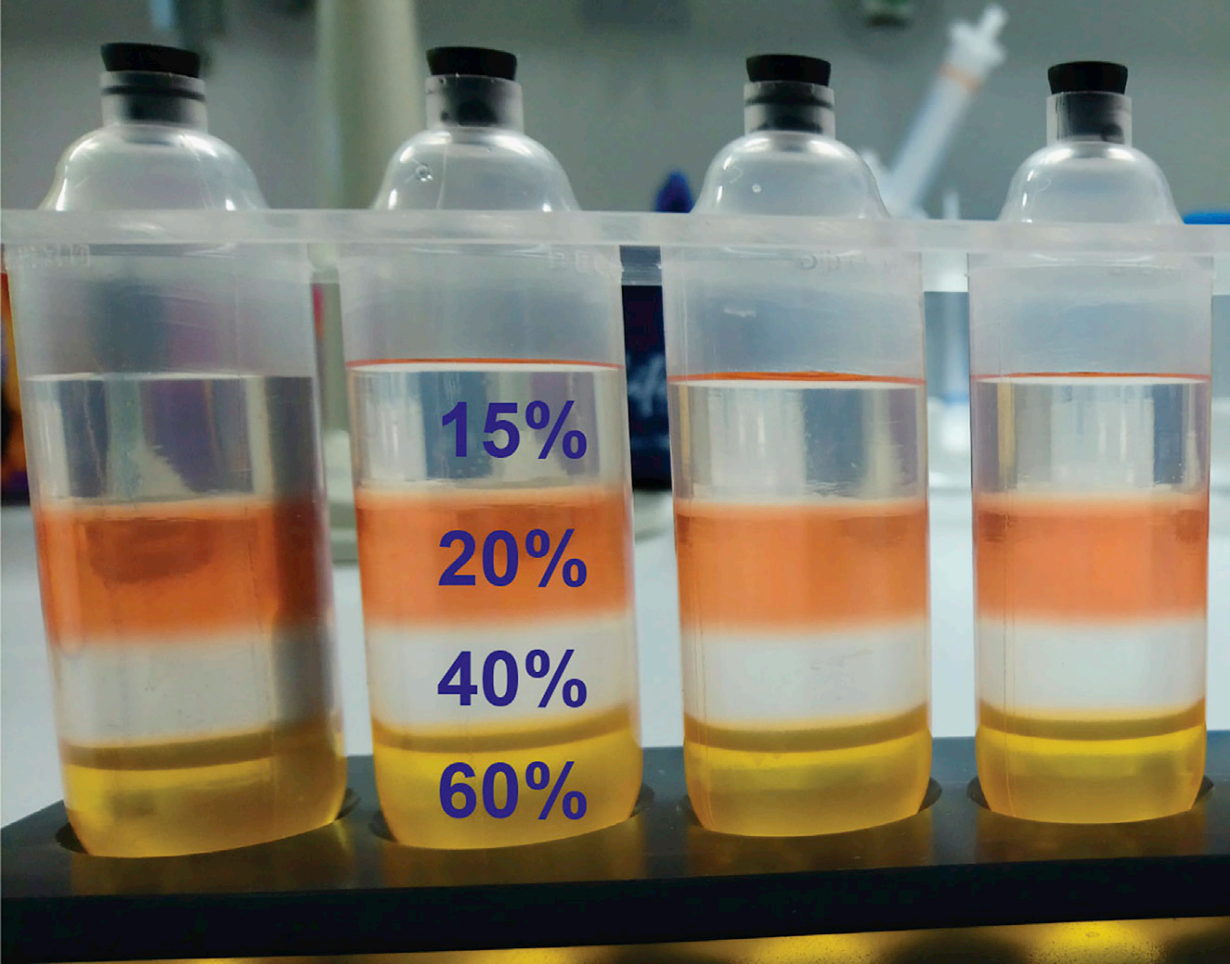
Discontinuous, Multilayered Iodixanol Density Gradients for AAV Isolation 15%, 20% 40% and 60% iodixanol solutions are underlaying in an ultra-centrifuge tube and should look as depicted in the photograph.

13. Prepare two multi-layered, discontinuous density gradients per AAV donor vector by underlaying iodixanol solutions of different concentrations (15%, 25%, 40%, and 60% (see Materials and Equipment)) in a 29.9 mL Beckman Opti-Seal ultra-centrifuge tube as depicted in Figure 1 (Cabanes-Creus et al., 2019).

a) First, add 6 mL of the 15% iodixanol solution to the bottom of the Beckman tube using a 10 mL syringe and a long 14-gauge metal needle.
b) Subsequently, underlay 6 mL of the 25% iodixanol solution, by placing the syringe needle into the bottom of the Beckman tube and gently elute the solution. Do not puncture the bottom of the tube.
c) Continue the same way with 5 mL of the 40% iodixanol solution and 5 mL of the 60% iodixanol solution, respectively.

**CRITICAL:** Air bubbles inside the syringe will reach the tube and disrupt the gradient. We recommend tapping the syringe, releasing the iodixanol solutions slowly and planning for enough dead volume.

**Figure 2:**
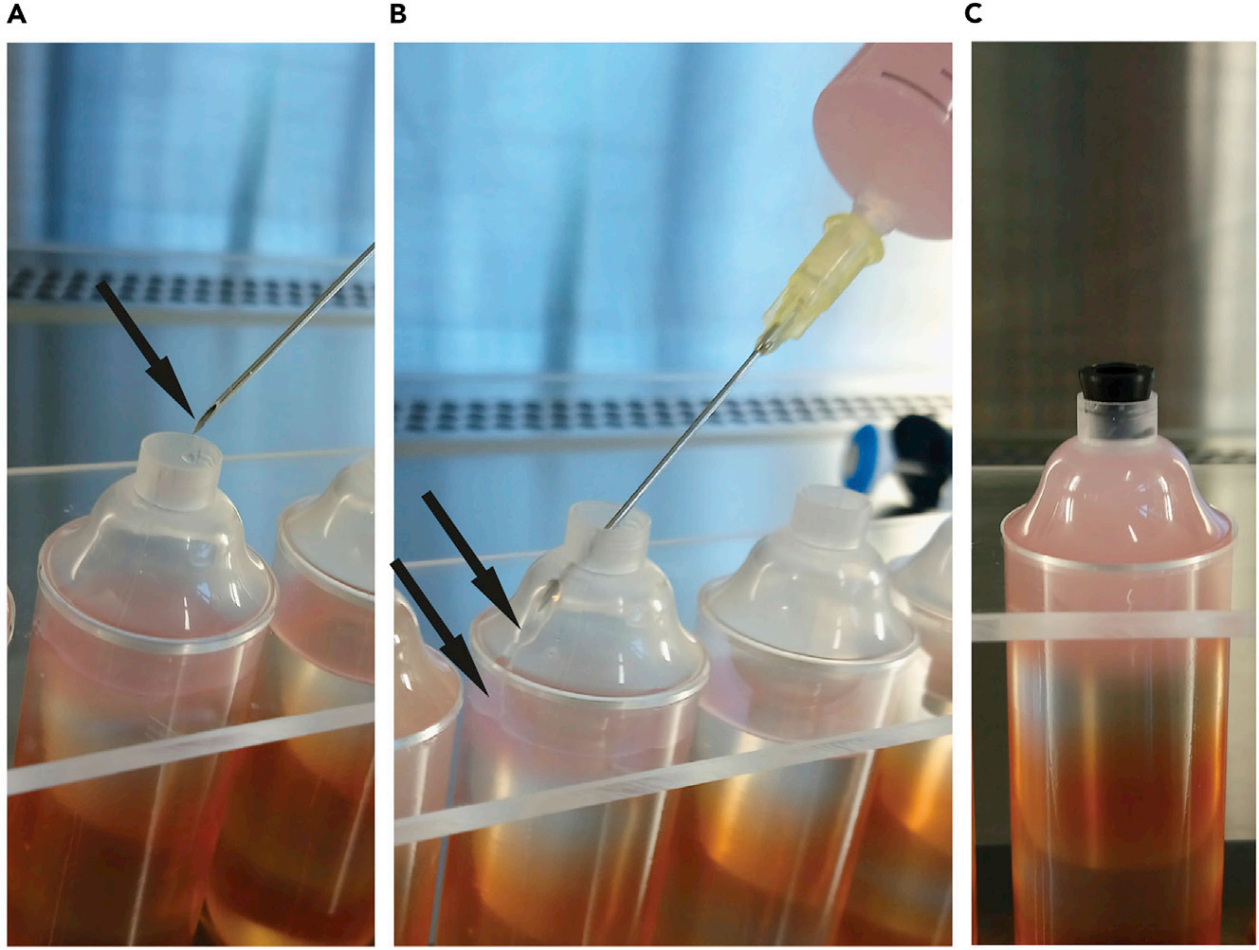
Loading Cell Lysate onto Density Gradients for Ultra-centrifugation (A and B) (A) Cell lysate is loaded on the ultra-centrifuge tube by establishing a flow on the tube wall. Face the bevel up, hold it close to the tube wall and (B) slowly load the cell lysate along the tube wall. (C) Balance the tubes with balancing buffer and seal it with the black cap.

***Note:*** ~15 mL of the AAV supernatant (step 12 above) can be split and loaded equally onto 2 gradient tubes. Fill up the Beckman tube with balancing buffer to the top of the tubes (Figure 2C) and balance to within 0.01 g difference.

**Pause Point:** Gradients can be prepared in advance and stored at 4°C for 15–18 h.

14. Use a 20-gauge needle to add the cell lysate to the iodixanol gradient tube by dripping slowly onto the top layer of the gradient (Figure 2A). To avoid disrupting the gradient, establish a flow along the tube wall as depicted in Figure 2B. Position the bevel of the needle upwards next to the cap of the tube.
15. Centrifuge at 345,322 × g in a Beckman Type 70Ti rotor for 2 h 10 min at 18°C.
16. Carefully carry the rotor to a sterile hood and remove the tubes with sterilized forceps.
17. Prepare a 10 mL syringe with an 18-gauge needle for extracting the AAV-containing 40% iodixanol layer.
18. Carefully clean the tubes with ethanol and a Kimwipe tissue before puncturing the Opti-Seal tube.

**Figure 3:**
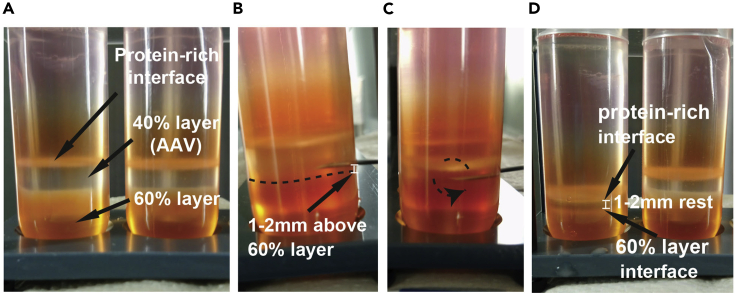
Extraction of the AAV-Containing Layer from the Ultra-centrifuge Tube (A) Appearance of the different iodixanol gradient after ultracentrifugation. (B) Insertion of the needle (bevel face up) 1–2 mm above the 60% iodixanol layer. (C) The remaining volume of the layer is extracted with the bevel face down to prevent contamination with the protein-rich layer. (D) Endpoint of extraction (right) compared to before extraction (right).

***Note:*** After ultra-centrifugation, different fractions of the cell lysate are separated in different iodixanol gradient layers. Proteins and cell debris accumulate in 25%-40% interface. The 40% iodixanol layer contains the AAV particles (Figure 3A).

19. Carefully insert the needle with the bevel up approximately 1–2 mm above the 60% gradient layer to collect the 40% gradient layer containing AAV particles (Figure 3B).
20. Remove the black cap before extracting the AAV layer.
21. Gradually withdraw 3–5 mL of the AAV-containing layer. After collecting half of the layer, turn the needle with bevel down to collect the remaining layer (Figure 3C).

**CRITICAL:** Be careful during handling and avoid disrupting the gradient layers. Do not collect the protein-rich interface white band at the interface of 25% and 40% layers. To avoid contamination with cell debris or proteins or iodixanol of the 60% iodixanol layer, the 40% AAV-containing layer should not be collected entirely (Figure 3D).

**Pause Point:** The AAV fraction can be stored at 4°C for several days, if necessary. For longer storage, store the AAV fraction at -80°C.

### Dialysis of AAV-Containing Iodixanol Solution and AAV Concentration

**Timing: 2 days**

Dialyze the AAV solution to remove remaining iodixanol and concentrate AAV particles.

***Note:*** All centrifuge steps are carried out at 2,900 × g.

**Figure 4:**
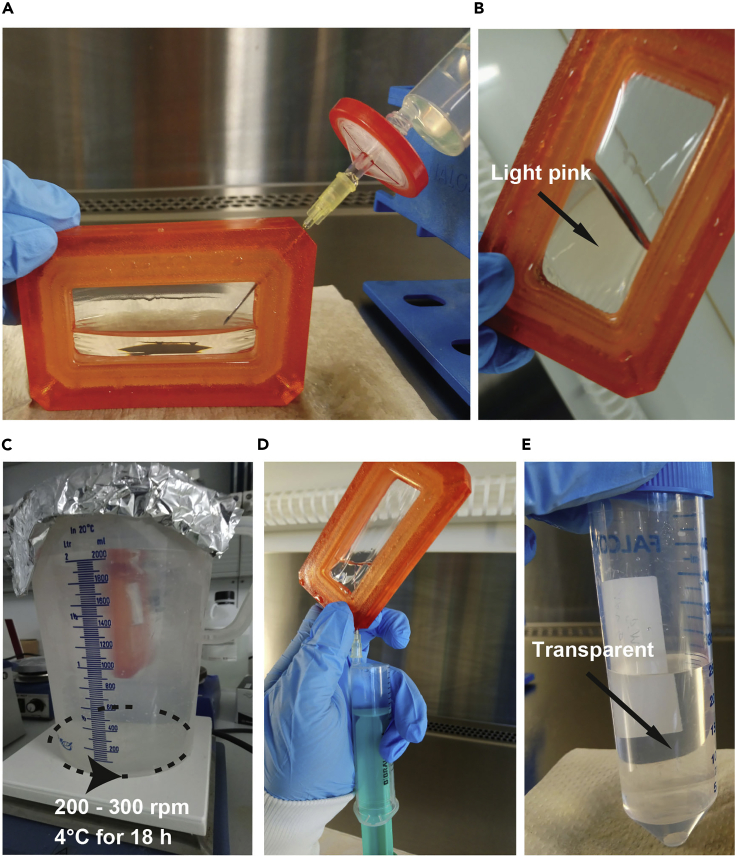
Dialysis of the Extracted AAV-Containing Iodixanol Layer (A) Injection of the extracted AAV solution from ultra-centrifuge tubes. (B) The loaded viral solution appears in a light pink color. (C) The loaded cassette is incubated for 18 h in cold dialysis buffer and magnetically stirred at 200–300 rpm to exchange iodixanol with dialysis buffer. (D) Extraction of the cleared AAV-containing solution from the cassette: Hold the cassette vertical to extract the remaining solution from the corner of the dialysis cassette. (E) The extracted solution is transferred to a new 50 mL falcon and should appear transparent.

22. Prime a 10 k MWCO dialysis cassette by placing the cassette into a container containing 2 L of dialysis/concentration buffer for 1 minute.
23. Pool AAV extractions from two gradient tubes (~10 mL in total) and filter the AAV solution through a 0.45 μm PES membrane filter directly into the primed MWCO dialysis cassette (step 22 above) as depicted in Figure 4A. Before dialysis, the AAV supernatant has a light pink color (Figure 4B).

24. Transfer the AAV-loaded dialysis cassette to the dialysis container and dialyze at 4°C for 15–18 h with gentle magnetic stirring at 250–300 rpm (Figure 4C).
25. Use a 20 mL syringe with a 20-gauge needle to collect the AAV solution in the dialysis cassette and transfer it to a 50 mL collection tube (Figures 4D and 4E). Now, the extract from the dialysis cassette should appear transparent (Figure 4E).

**CRITICAL:** Be careful handling the dialysis cassette and avoid destroying the dialysis membrane.

***Note:*** If you observe debris in the dialyzed AAV solution, filter again through a 0.45 μm PES syringe filter before starting concentration steps. The maximum capacity of the dialysis cassette is 12 mL.

**Pause Point:** After filtration, the dialyzed AAV solution can be stored at 4°C for concentration on the next day.

26. Prime a 100 K Amicon Ultra-4 centrifuge filter by adding 15 mL of dialysis/concentration buffer to the filter and centrifuge for 5–10 min. Discard the flow-through.
27. Load up to 15 mL of the dialyzed AAV supernatant to dialysis/concentration buffer-primed 100 K Amicon Ultra-4 centrifuge filter and centrifuge for 10–15 min. Discard the flow-through and load the remaining AAV supernatant onto the same Amicon Ultra-4 filter, fill up to 15 mL with dialysis/concentration buffer and centrifuge for 10–15 min. Discard the flow-through.
28. Wash the AAV particles on the membrane of the Amicon Ultra-4 filter with 15 mL of dialysis/concentration buffer and centrifuge for 10–15 min. Discard the flow-through and repeat the washing step two more times.
29. Use a 1000 μL pipette to collect the remaining AAV solution (~200–500 μL) in the concentrating filter. Pipette up and down several times and transfer the AAV solution to a 1.5 mL Eppendorf tube.
30. Aliquot the AAV solution in smaller volumes (50–100 μL) and store it at -80°C for long-term storage. Take a 5 μL AAV aliquot for titration using real-time PCR.

***Note:*** To collect remaining AAV particles, we recommend adding 500–1000 μL fresh dialysis/concentration buffer to the concentrating filter, pipette up and down and transfer the AAV solution to the AAV collection tube (step 29 above).

**Pause Point:** The 5 μL aliquoted AAV can be frozen at -80°C before performing real-time PCR.

### Titration of AAV Particles by Real-Time PCR with TaqMan Probes

**Timing: 3–4 h**

Prepare standard curve using AAV genome plasmid and titrate AAV particles by TaqMan real-time PCR.

31. Prepare a standard curve using your plasmid stock and adjust it to 2 × 10^9^ molecules/μL

Example calculation:

We assume that the average molecular weight for a DNA base pair is 650 Da.
Plasmid Size (pAAV-Lmnb1-T2A-mCherry): 7216 bp
Plasmid Concentration: 1 μg/μL
Plasmid Molecular Weight: 7216 bp × 650 Da/bp (g/mol) = 4.69 × 10^6^ g/mol
Plasmid (mol/μL): 1 (μg/μL) × 1 (g)/1 × 10^6^ (μg) × 1/4.69 × 10^6^ (g/mol) = 2.13 × 1 × 10^−13^ (mol/μL)
Plasmid (molecules/μL): 2.13 × 1 × 10^−13^ mol/μL × (Avogadro constant) 6.022145 × 1 10^23^ (molecules/mol) = 1.28 × 10^11^ molecules/μL
Prepare 100 μL of plasmid with 2 × 10^9^ molecules/μL:
Dilution factor: 1.28 × 10^11^ (molecules/μL)/2 × 10^9^ (molecules/μL) = 64.2X dilution
Thus, dilute 1.56 μL plasmid stock with 98.4 μL water (100 μL/64.2=1.56 μL).
32. Prepare a serial 10-fold-stock dilution for the plasmid: 2 × 10^8^, 2 × 10^7^, 2 × 10^6^, 2 × 10^5^, 2 × 10^4^ molecules/μL to create a standard curve by real-time PCR.
33. Prepare DNase I working solution by adding 550 μL of water to 1 stock vial of DNase I.
34. Prepare a mixture as follows: 10 μL RDD buffer + 2.5 μL DNase I + 5 μL of AAV and fill up with water up to 100 μL total volume. Incubate at 37°C for 30 min. This will be a 1/20 dilution of the AAV.
35. Serially dilute the AAV 5-fold with water for real-time PCR: 1/100, 1/500, 1/2500 and 1/12500.
36. Prepare a TaqMan real-time PCR reaction as depicted in Table 1.

37. Run the PCR reaction following Table 2.

**Table 1:** Reagents of a Single 20 μL TaqMan Real-Time PCR Reaction

Reagents	Final Concentration	Volume (μL)
AAV-ITR Forward (10 μM)	0.3 μM	0.6
AAV-ITR Reverse (10 μM)	0.3 μM	0.6
AAV-ITR TaqMan Probe (10 μM)	0.25 μM	0.5
2X TaqMan Universal PCR MasterMix	1x	10
AAV or plasmid serial dilutions	n/a	5
Dnase/Rnase-free water	n/a	3.3
**Total**	**n/a**	**20**

38. AAV titers are calculated as viral genome copies per μL as described in section QUANTIFICATION AND STATISTICAL ANALYSIS.

**CRITICAL:** The standard curve is essential for accurate calculation of AAV titer. Be careful preparing serial dilution of the AAV genome plasmid.

***Note:*** Treatment of the AAV stock with DNase I is necessary to degrade residual DNA plasmids in the AAV solution. Run your samples and standard at least in duplicates and include non-template controls (master mix without templates or water).

**Table 2:** PCR Condition for TaqMan Real-Time PCR

Step	Temperature	Time
1	50°C	2 minutes
2	95°C	10 minutes
3	95°C	15 seconds
4	60°C	1 minutes
5	Go to step 3 for 39 more times	

### Isolation and Culture of Mouse HSPCs

**Timing: 3–5 h**

Isolate, enrich Sca1^+^ cells from mouse bone marrow using MACS separation and culture isolated HSPCs *in vitro*.

***Note:*** All centrifuge steps are carried out at 400 × g for 7 min at 4°C.

39. To isolate Sca1^+^ cells from mouse bone marrow (BM), dissect left and right femurs and tibias from 8–12 weeks old mice. Remove muscles and connective tissue and transfer femurs and tibias to a well of 6-well plate containing 3 mL of FACS buffer.
40. Use a 1 mL syringe and a 25-gauge needle to flush out the BM from the end of femurs and tibias, repeat this step several times until the bones become white. Transfer the BM cell suspension to a 15 mL collection tube and wash the well one time with 5 mL of FACS buffer. Centrifuge the BM cell suspension.
41. Discard supernatant and resuspend the BM cell pellet with 2 mL of red blood cell lysis buffer. Pipette up and down 10 times.
42. Incubate on ice for 5 min, subsequently add 10 mL of FACS buffer and centrifuge the BM cell suspension.
43. Discard supernatant, resuspend the BM cell pellet with 2 mL FACS buffer and mix well by pipetting up and down 10 times. Count total BM cell number using a hemocytometer before magnetic cell separation (MACS).

***Note:*** We routinely obtain ~4 × 10^7^ total BM cells from 2 femurs and tibias per mouse. The following steps of MACS Sca1^+^ enrichment are described for a maximum 4 × 10^7^ total BM cells; if working with larger cell number, scale up all reagents accordingly. Try to keep cells and solutions on ice.

**Pause Point:** the BM cell suspension can be kept on ice for 1–2 h before proceeding MACS enrichment.

**Figure 5:**
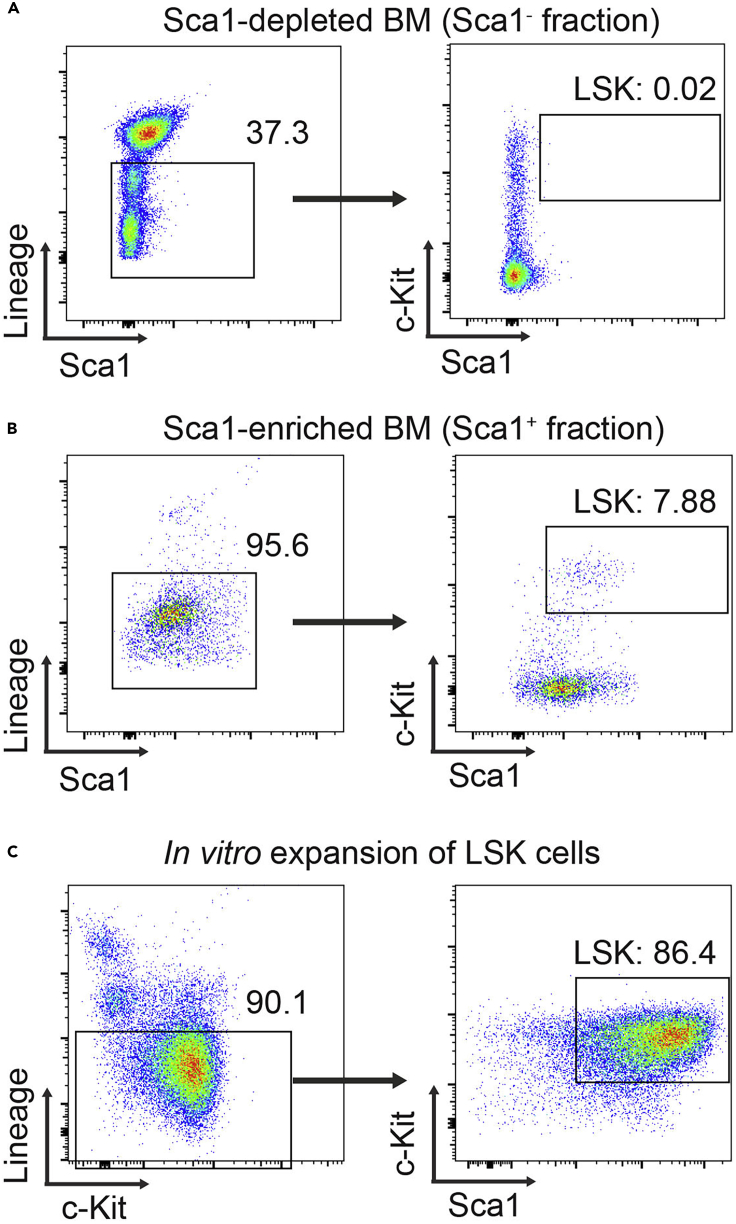
FACS Plots Showing Lin^−^Sca1^+^c-Kit^+^ (LSK) Cell Fractions after MACS Purification and 2 Days of Culture Bone marrow cells were stained with α-mouse lineage cocktail, Sca1, and c-Kit antibodies. (A) The negative fraction of the LS column should be almost completely depleted of Sca1^+^ cells. (B) The positive fraction eluted from the LS or MS separation column is enriched in LSK cells and progenitor cells, which all express Sca1. The LSK population should comprise 6 to 8% of the Sca1^+^ cell fraction. Lymphoid progenitor cells require IL-7 to survive and will be depleted upon culturing with 50 ng/mL mTPO, mSCF, mFLT-3, and hIL-11. (C) Purified LSK cell fraction after 2 days of culture in HSPC medium.

44. Centrifuge the BM cells and discard the supernatant.
45. Resuspend the BM cell pellet in 382 μL FACS buffer, add 10 μL Fc blocking antibody (α-mouse CD16/32) to the BM cell suspension, and incubate for 10 min on ice.
46. Add 8 μL α-mouse Sca1-FITC antibody to the BM cell suspension and mix well by pipetting up and down 10 times. Incubate the Sca1-FITC-labeled BM cells for 10 min in the dark on ice.
47. Wash the Sca1-FITC-labeled BM cells one time by adding 10 mL of FACS buffer and centrifuge.
48. Discard supernatant, resuspend the Sca1-FITC-labeled BM cells in 320 μL FACS buffer and add 80 μL α-FITC MicroBeads to the cell suspension. Mix well by pipetting up and down several times and incubate for 15 min at 4°C in the refrigerator. Mix the cell suspension every 5 min during incubation by brief vortexing.
49. Wash cells by adding 10 mL of FACS buffer and centrifuge. Discard the supernatant and resuspend the BM cell pellet in 500 μL of FACS buffer.
50. Insert a LS separation column with the column wings to the front into a MACS separator, insert a 50 μm sterile BD cup-type filcon on top of the LS column. Rinse the LS column by applying 3 mL of FACS buffer through the BD cup filcon to the LS column and let FACS buffer run through. Load 500 μL of the BM cell suspension through the BD cup filcon into the prepared LS column and then wash the BD cup filcon two times with 500 μL FACS buffer. Collect the total effluent (which corresponds to the unlabeled (Sca1^−^) cell fraction) into a 15 mL falcon tube and set aside for determining the purity of MACS separation later.
51. Remove the BD cup filcon and wash the LS column 3 times by adding 3 mL FACS buffer each time once the LS column reservoir is empty.
52. Remove the LS column from the MACS separator and place it on a 15 mL collection tube. Pipette 5 mL FACS buffer onto the LS column and immediately flush out the magnetically labeled cells (Sca1^+^) by firmly applying the plunger which is supplied with the column.
53. Count the cells with a hemocytometer.
54. Determine the purity of Sca1^+^ cell fraction. Stain the BM cells before MACS as well as the Sca1^−^ and Sca1^+^ cell fractions with α-mouse lineage antibody cocktail, c-Kit and Sca1 antibodies for 15 min at 4°C. Wash the stained cells with FACS buffer and analyze them using FACS analyzer and FlowJo software (Figures 5A and 5B).

55. Centrifuge the Sca1^+^ cells and discard the supernatant. Resuspend the Sca1^+^ cell pellet in serum-free HPSC medium (StemSpan™ SFEM II medium supplemented with mouse SCF (50 ng/mL), mouse TPO (50 ng/mL), mouse Flt3-L (50 ng/mL) and human IL-11 (50 ng/mL)) at a density of 2 × 10^5^ cells/mL. Culture 4 × 10^5^ Sca1^+^ cells in a well of 6-well plate in total volume of 2 mL.
56. The next morning, passage the Sca1^+^ cells by transferring a half of the volume (1 mL) to a well of 6-well plate and add 1 mL HSPC medium to reach 2 mL per well.
57. Determination of the proliferation rates of cultured HSPCs (optional)
a) Wash 1 × 10^6^ Sca1^+^ cells (step 53 above) two more times by adding 10 mL of PBS and centrifuge. Resuspend the cell pellet in 1 mL of PBS, add CellTrace violet to a final concentration of 5 μM and pipette up and down 10 times. Label these cells at 37°C for 15 min in the dark.
b) Wash the labeled cells by adding 10 mL of serum-free StemSpan™ SFEM II medium and centrifuge.
c) Follow steps 55 to 56 above for culturing and passaging mouse HSPCs in HSPC medium.
d) After 48 h of CellTrace labeling, the proliferative activity of the cultured HSPCs is determined by flow cytometry.

**CRITICAL:** It is important to maintain mouse HSPCs at low density (≤ 2 × 10^5^/mL) to obtain high HR efficiency and high survival rates.

***Note:*** In order to increase the efficiency of Sca1^+^ MACS enrichment, we recommend repeating the magnetic separation procedure from steps 49 to 52 with a new column. Depending on cell yield, the eluted fraction from step 52 should be further enriched over a MS (smaller capacity) or a LS column (larger capacity).

**Pause Point:** After passaging, the cultured HSPCs are placed back into cell incubator for further 24 h.

### CRISPR/Cas9/AAV-Mediated HR in Mouse HSPCs

**Timing: 2–3 h**

Insert the coding sequences of the self-cleavage peptide T2A coupled to the fluorescent marker mCherry (T2A-mCherry) in-frame into the last exon of the mouse Lamin B1 (*Lmnb1*) gene in mouse HSPCs.

***Note:*** All centrifuge steps are carried out at 400 × g for 5 min at 22°C–24°C.

58. Collect the activated HSPCs by pipetting up and down 10 times and transfer to a 15 mL collection tube after 48 h of culture. Determine the cell concentration using a hemocytometer and centrifuge the activated HSPCs. Discard the supernatant and wash the activated HSPCs one time with 22°C–24°C PBS by resuspending in 10 mL PBS and centrifuge. Remove the supernatant and resuspend the cell pellet in Amaxa electroporation buffer at a density of 2–3 × 10^5^ HSPCs per 20 μL. Follow step 59 for HSPCs isolated from Cas9 transgenic mice or step 60 for isolated from C57BL/6 mice.
59. **Gene editing in Cas9 transgenic-derived HSPCs:** due to constitutive Cas9 expression, only the delivery of sgRNA is required for gene targeting.
a) Transfer 20 μL cell suspension in Amaxa electroporation buffer (total 2–3 × 10^5^ cells) in step 1 to a 1.5 mL Eppendorf tube.
b) Add 100 pmol sgRNA to the cell solution. Mix well by pipetting up and down 10 times and continue with step 61 below.
60. **Gene editing in C57BL/6-derived HSPCs:** sgRNA/Cas9 ribonucleoprotein (RNP) complexes are required for gene editing.
a) Per reaction/electroporation, mix 50 pmol wild-type SpCas9 protein (from IDT or produced in-house) with 100 pmol sgRNA (Synthego or IDT), and incubate 10 min at 22°C–24°C.
b) Transfer 20 μL cell suspension in Amaxa electroporation buffer (total 2–3 × 10^5^ cells) in step 1 to a 1.5 mL Eppendorf tube.
c) Add CRISPR/Cas9 RNP complexes to the cell suspension. Mix well by pipetting up and down 10 times and continue with step 61 below.
61. Transfer 20 μL electroporation mixture to a well of a 16-well Nucleocuvette strip.
62. Electroporate the cassette in the Amaxa™ 4D-Nucleofector™ with the “mouse B cell program” provided by the manufacturer.
63. Add 80 μL of pre-warmed HSPC medium to the well of the electroporation cassette to collect electroporated HSPCs. Transfer the electroporated HPSCs to a well of a 12-well plate containing 1 mL pre-warmed HSPC medium.
64. Transfer 10^4^ electroporated HSPCs to a well of a 48-well plate containing 500 μL HSPC medium. 30 min later, infect the electroporated HSPCs with AAV-DJ carrying the donor template at a MOI of 5 × 10^6^ genome copies per cell. Place AAV-infected HSPCs back into the incubator.
65. Two days post editing, collect the targeted HSPCs by pipetting up and down, pool together into a 15 mL collection tube and count cell number with a hemocytometer. 20% of total targeted HSPCs are saved for analyzing HR efficiency by flow cytometry (continue step 66 below) or PCR amplification and sequencing (continue step 67 below). The remaining cells can be used for transplantation to assess engraftment efficiency of the targeted HSPCs *in vivo.*

**CRITICAL:** To minimize cell death due to electroporation, do not use expired electroporation buffer. The AAV volume should not exceed 20% of the total volume.

***Note:*** Depending on the experimental set up, the number of the electroporated HSPCs infected with AAV donor vectors can be scaled up accordingly.

***Note:*** After 48 h of culture in HSPC medium, mouse HSPCs should have increased in size and formed cell clusters indicating proliferation. During establishing gene knockin experiments in mouse HSPCs, try to use different AAV MOI numbers in order to optimize HR efficiency. Include the following controls for testing HR efficiency: 1) only electroporated, 2) only infected with AAV-DJ, and 3) only sgRNA-electroporated cells without AAV-DJ.

**Pause Point:** For quantification of HR and NHEJ events by PCR amplification and sequencing, 20% of the total targeted HSPCs are collected by centrifuging and the cell pellet can be stored at -20°C for several days before extracting genomic DNA.

### Evaluation of HR Efficiency

**Timing: 2–48 h**

Determine HR efficiency by flow cytometry (mCherry^+^ cells) or quantify of HR and NHEJ events in the targeted *Lmnb1* locus by PCR amplification and sequencing.

***Note:*** All centrifuge steps are carried out at 400 × g for 5 min at 4°C.

66. Determination of HR efficiency by flow cytometry:
a) 20% of the total targeted HSPCs (step 65 above) are transferred to a FACS tube and centrifuged.
b) Discard the supernatant, wash the targeted HSPCs with 1 mL PBS and centrifuge. Discard the supernatant, resuspend the HSPCs in 100 μL FACS buffer containing Fc blocking antibody (α-mouse CD16/32, diluted 1:250) and incubate at 4°C–8°C for 10 min.
c) Add the stem cell antibody staining cocktail (α-mouse lineage-cocktail, c-Kit, Sca1, CD48 and CD150) to the FACS tube and incubate at 4°C–8°C for 15 min.
d) Add 1 mL FACS buffer to the FACS tube and centrifuge. Discard the supernatant and wash the cells by adding 1 mL FACS buffer to the FACS tube. Centrifuge the cells and discard the supernatant. Finally, resuspend the stained HSPCs in 200–300 μL FACS buffer containing 1 μg/mL DAPI for flow cytometric analysis.
e) The stained HSPCs are analyzed in a BD Fortessa analyzer with the flow rate setting in slow mode.
f) Export and analyze the raw data using FlowJo software. Frequencies of mCherry^+^ cells are determined in all cell fractions (Lineage (Lin)^−^Sca1^+^c-Kit^+^ (LSK), HSC/multipotent progenitor (MPP)1, MPP2, MPP3/4).
67. Quantification of HR and NHEJ events by PCR amplification and sequencing:
a) 20% of the total targeted HSPCs (step 65 above) are transferred to a 1.5 mL Eppendorf tube and centrifuged.
b) Discard the supernatant and wash the cells by adding 1 mL PBS to the collection tube. Centrifuge the cells and discard the supernatant. Resuspend the cell pellet in QuickExtract™ DNA Extraction Solution at a density of 10^4^ cells/10 μL.
c) Mix well and briefly vortex to completely lyse cells. Transfer a maximum of 100 μL lysed cells into a PCR tube.
d) Denature genomic DNA by following thermal cycle: 65°C for 15 min, 68°C for 15 min, 95°C for 15 min and 4°C using a PCR machine. The denatured genomic DNA is ready for PCR amplification.
e) Perform PCR amplification using primer sets binding outside of the HAs (see step 3 in section Before You Begin) and LongAmp PCR master mix or alternative DNA polymerases (KOD hot start DNA polymerase) for long-range PCR.
f) Run PCR products on 1%–2% agarose gel and take a photograph. HR bands are larger than the NHEJ or wild-type (WT) bands which have similar size.
g) Band quantification can be performed using the free ImageJ software (Schneider et al., 2012). HR efficiency is estimated on the basis of band intensity.
h) HR and NHEJ/WT bands are extracted from the gel and cloned into pJET plasmids using the CloneJET PCR cloning kit. PCR colonies are sequenced by Sanger sequencing. Basing on the nucleotide sequences of the targeted loci, the relative frequencies of HR, NHEJ and WT events are determined.

## EXPECTED OUTCOMES

Using this step-by-step protocol, we routinely produce up to total 8 × 10^13^ viral genome copies from eight 150-mm dishes of transfected HEK293T cells. For long-term storage, we recommend that the AAV solution should be aliquoted into 50–100 μL volumes and stored at -80°C. After thawing, the remaining AAV solution can be stored at -20°C for one month.

The purity of the Sca1^+^ cell fraction after MACS enrichment should be ~95%–97%. ~10% of the cells in the Sca1^+^ cell fraction are LSK cells. Two days post stimulation with stem cell cytokines, the LSK cell compartment should have expanded to up to 80%–90% (Figure 5C). Normally, we achieve up to 1 × 10^6^ LSK cells isolated from a mouse after 2 days *in vitro* culture.

In our hands, this step-by-step protocol for gene knockin and gene repair in mouse HSPCs routinely achieves efficient HR (~25%–30%) in several gene loci by using CRISPR/Cas9 in combination with AAV-DJ to deliver the donor templates. Screening other AAV serotypes might help to further increase HR efficiency in mouse HSPCs. In addition, the inhibition of the NHEJ pathway by small inhibitors might also improve HR efficiency in mouse HSPCs.

Our system is suitable to model blood disorders that are caused by mutations in more than one gene. Our protocol allows the simultaneous targeting of multiple gene loci in mouse HSPCs. In Tran et al. (2019), we inserted in-frame coding sequences of T2A-mCherry and T2A-BFP reporters into the last exon of the *Lmnb1* and beta-actin (*Actb*) genes, respectively. We obtained approximately 3.5% of double-positive LSK cells expressing both mCherry and BFP reporters.

Upon transplantation into irradiated immunodeficient mice, the targeted HSPCs engraft efficiently and fully reconstitute the hematopoietic system with all immune cell lineages detected in the bone marrow and spleen of the transplanted animals at 8- or 16-weeks post reconstitution (see Tran et al., 2019).

## QUANTIFICATION AND STATISTICAL ANALYSIS

### Calculation of AAV Titers

We typically use the following procedure to calculate AAV titers in viral genome copies per μL using the standard curve.

**Figure 6:**
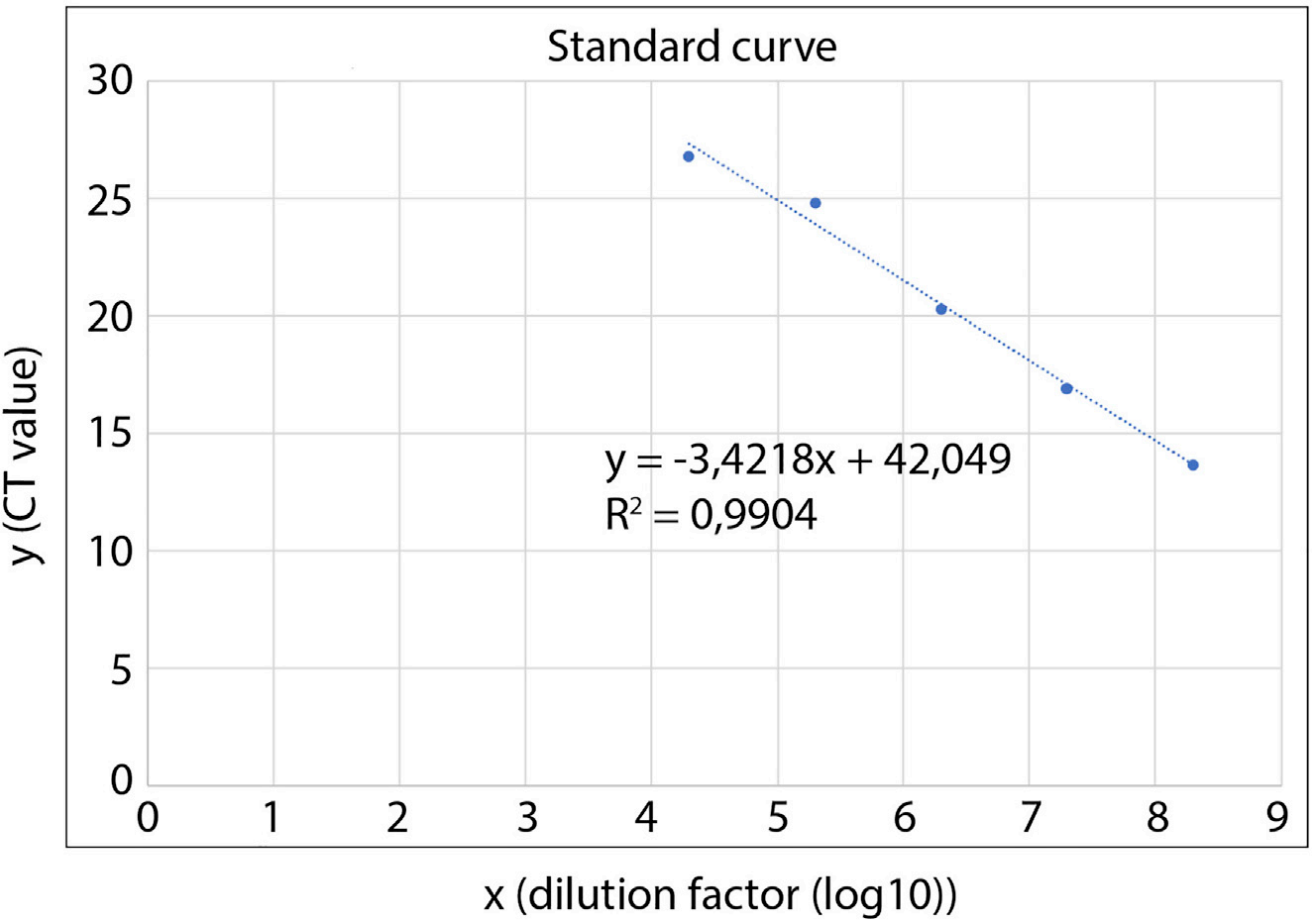
Generation of Standard Curve for Calculating AAV Titer Using TaqMan Real-Time PCR CT values from serial dilution of the AAV genome plasmid are represented on the y-axis. The dilution factor of the diluted plasmid is represented as log10 value on the x-axis.

1. Calculate the average of the CT-value of each plasmid and AAV-dilution from step 38.
2. Prepare a standard curve from the 5 plasmid dilutions.
3. Calculate a linear regression (y=mx+c) and determine the coefficient of determination (R^2^). R^2^ should be >0.98. An exemplary graph is shown in Figure 6.

4. Use slope (m) and intersection of the y-axis (c) to calculate x=(y-c)/m. Y is the average CT of each AAV-dilution.
5. Example according to values given in Figure 6: x= (averageCT- 42,049)/-3,4218
6. Calculate 10^x^ for each serial AAV-dilution.
7. Multiply the value by the dilution factor for each serial AAV-dilution.
8. Average all values to calculate the real AAV-titer.

## LIMITATIONS

One important limitation of this protocol is the maximal AAV packaging capacity of 4.5 kb. However, within these limits we successfully performed targeted insertions of 0.8 kb reporters into the *Lmnb1* and beta-actin (*Actb*) loci and replaced a neomycin cassette of 2 kb with a Rag2 wild-type sequence of 0.8 kb. Furthermore, maximal HR in HPSCs is dependent on cellular proliferation activity, because only cells in S and G2 phase preferentially use the HR pathway. Enriched mouse Sca1^+^ cells are a heterogeneous population including true stem cells and various differentiating progenitor cell subsets. In our culture conditions, the HSC and MPP1 LSK subsets proliferate more slowly than the more differentiated MPP2 and MPP3/4 LSK subsets. As a result, HR efficiency in the HSC and MPP1 subsets is lower (~10%) compared to the MPP2 and MPP3/4 subsets (up to 30%) (Tran et al., 2019).

## TROUBLESHOOTING

### Problem

Low quality of purified AAV donor vectors can lead to low HR efficiency and low survival of mouse HSPCs. Furthermore, AAV infection causes induction of cell death and proliferation arrest in mouse HSPCs.

### Potential Solution

Low quality of purified AAV batches may be due to unhealthy HEK293T cells, transfection failure or incomplete HEK293T cell lysis. First, low passage (<15 passages) mycoplasma-free HEK293T cells should be used. Second, the transfection efficiency of the three plasmids (AAV production, step 3) should be regularly assessed with a pAAV-CMV-GFP-reporter genome plasmid as a standard. Third, the transfected HEK293T cells must be completely lysed in order to obtain high amounts of AAV particles. Lysis efficiency can be monitored in parallel to an experiment with pAAV-CMV-GFP transfected HEK293T cells as the supernatant turns light green after three freeze/thaw cycles. We recommend to carefully determine the AAV titers by real-time PCR and to test each AAV batch with mouse HPSCs for efficient HR and low toxicity. Notedly, the AAV volume is kept below 20% of the total culture volume.

### Problem

Contamination of residual DNA plasmids in the purified AAV solution may negatively influence the survival of mouse HSPCs and results of AAV titration by real-time PCR.

### Potential Solution

In order to remove residual DNA plasmids in the purified AAV solution, the amount of Benzonase (per 1 mL cell lysate) should therefore be scaled according to the number of 150-mm dishes combined into one 15 mL collection tube (step 5). We recommend adjusting the Benzonase units per mL of the cell lysate as follows: 2 dishes: 50 U/mL, 4 dishes: 100 U/mL or 8 dishes: 200 U/mL. In addition, digesting time with Benzonase can be carried out at 37°C for 2 h.

### Problem

Low survival of the electroporated HSPCs can be caused by expired electroporation buffer.

### Potential Solution

To avoid this problem, the electroporation buffer must be filtered before use and stored no longer than 3 months at 4°C. In addition, screening of different electroporation programs might be useful to enhance the gene editing efficiency.

### Problem

Low HR efficiency due to low proliferative activity and quality of the activated HSPCs.

### Potential Solution

The HR pathway is active during the S and G2 phases of the cell cycle. In order to maximize HR efficiency, we thus recommend that before performing knockin experiments the proliferative activity of mouse HSPCs should be monitored by flow cytometry (see step 57). The Sca1^+^ cell fraction obtained by MACS contains only 10% LSK cells that respond to stem cell cytokines, while 90% of the Sca1^+^ progenitor cells remain unresponsive, undergo apoptosis and become toxic to neighboring LSK cells. Therefore, we recommend using a dead cell removal kit (Miltenyi Biotec) during HSPC culture (Steps 55 to 56). In addition, we suggest that mouse HSPCs are cultured at a low density of 2 × 10^5^ cells/mL to minimize toxicity from dying cells.
